# A phase II randomized trial comparing radiotherapy with concurrent weekly cisplatin or weekly paclitaxel in patients with advanced cervical cancer

**DOI:** 10.1186/1748-717X-5-84

**Published:** 2010-09-23

**Authors:** Fady B Geara, Ali Shamseddine, Ali Khalil, Mirna Abboud, Maya Charafeddine, Muhieddine Seoud

**Affiliations:** 1 Department of Radiation Oncology, The American University of Beirut Medical Center, Bliss Street, Beirut, Lebanon; 2 Department of Medical Oncology, The American University of Beirut Medical Center, Bliss Street, Beirut, Lebanon; 3 Department of Obstetrics and Gynecology, Division of Gynecologic Oncology, The American University of Beirut Medical Center, Bliss Street, Beirut, Lebanon

## Abstract

**Purpose/Objective:**

This is a prospective comparison of weekly cisplatin to weekly paclitaxel as concurrent chemotherapy with standard radiotherapy for locally advanced cervical carcinoma.

**Materials/Methods:**

Between May 2000 and May 2004, 31 women with FIGO stage IB2-IVA cervical cancer or with postsurgical pelvic recurrence were enrolled into this phase II study and randomized to receive on a weekly basis either 40 mg/m^2 ^Cisplatin (group I; 16 patients) or 50 mg/m^2 ^paclitaxel (group II; 15 patients) concurrently with radiotherapy. Median total dose to point A was 74 Gy (range: 66-92 Gy) for group I and 66 Gy (range: 40-98 Gy) for group II. Median follow-up time was 46 months.

**Results:**

Patient and tumor characteristics were similar in both groups. The mean number of chemotherapy cycles was also comparable with 87% and 80% of patients receiving at least 4 doses in groups I and II, respectively. Seven patients (44%) of group I and 8 patients (53%) of group II developed tumor recurrence. The Median Survival time was not reached for Group I and 53 months for group II. The proportion of patients surviving at 2 and 5 years was 78% and 54% for group I and 73% and 43% for group II respectively.

**Conclusions:**

This small prospective study shows that weekly paclitaxel does not provide any clinical advantage over weekly cisplatin for concurrent chemoradiation for advanced carcinoma of the cervix.

## Introduction

In many developing countries, cervical cancer remains a major public health problem with high overall incidence and higher frequency of advanced stage at diagnosis. Radiation therapy remains the main treatment modality for patients with advanced cervical cancer the results of which depend on disease stage, tumor volume, presence of involved lymph nodes, delivered radiation dose, treatment duration, hemoglobin level, and the optimal use of intracavitary brachytherapy [1-5]. Nodal involvement, particularly of paraaortic nodes, was reported to be the most important adverse prognostic factor, reducing survival by one-half. A series of controlled randomized studies have shown that the outcome of these patients can be improved by the use of concurrent chemoradiotherapy (CTRT) protocols [6-16]. Based on these trials, the National Cancer Institute issued a clinical alert stating that "strong consideration should be given to the incorporation of concurrent cisplatin-based chemotherapy with radiation in women who require radiation therapy for cervical cancer" [17,18]. At present, the integration of radiosensitizing cisplatin-based chemotherapy with local treatment is considered the accepted standard in the management of high-risk patients with carcinoma of the cervix [19-21].

Despite the use of concurrent CTRT, many patients continue to fail in the pelvis (20-25%) and at distant sites (10-20%), [6,16,21-23]. In addition, the use of cisplatin-based chemotherapy concurrently with RT has not been invariably effective. A study by the National Cancer Institute of Canada using weekly concurrent single agent cisplatin has shown no clinical benefit from this schedule [24]. These facts have stimulated interests in exploring other concurrent combinations with potentially more clinical effect. Paclitaxel is a taxane chemotherapy drug that was found to have significant activity in solid tumors especially epithelial ovarian cancer, lung, and breast cancer [25-28]. Preclinical studies have shown a radiosensitizing effect of paclitaxel in human cervical cancer cell lines [29,30]. It was also shown that this drug exerts a preferential cytotoxic activity in human cervical cancer cells with low Raf-1 kinase activity which makes it desirable to be used in conjunction with radiotherapy [30]. The clinical feasibility of concurrent RT and paclitaxel was tested in phase I trials and a maximum tolerated dose (MTD) of 50 mg/m2 per week concurrently with radiation therapy was established [31,32]. In addition, the clinical efficacy of paclitaxel has been tested in phase II and III studies for metastatic and recurrent cervical cancer with objective response rates ranging between 36 and 47% [33-35].

In this study, we examine the tumor response, treatment toxicity, and outcome of patients with locally advanced cervical cancer treated by concurrent radiation therapy and chemotherapy using either weekly Cisplatin or weekly paclitaxel.

## Methods and materials

### Patients

Patients presenting to the American University of Beirut Medical Center, with advanced carcinoma of the cervix, stages IB2-IVA according to the Federation Internationale de Gynecologie Obstetrique (FIGO) staging system, or with measurable central pelvic recurrence, were eligible to enroll in this phase II randomized prospective study. Inclusion criteria also included: age <80 years; Gynecologic Oncology Group (GOG) performance status of 0-3; adequate hematological and biochemical profile with absolute neutrophil count >1.5 × 10^9^/L, platelets >100 × 10^9^/L; creatinine <1.5, liver enzymes (AST and ALT) <3 × normal, and bilirubin <1.25 normal. Patients with evidence of enlarged paraaortic lymphnodes, history of peripheral neuropathy, prior radiotherapy, prior chemotherapy (neoadjuvant), hypersensitivity to cisplatin or paclitaxel, or other synchronous malignancies were considered not eligible. Treatment was started within 48 hours of randomization. Between May 2000 and May 2004, 31 women were enrolled and randomized to receive on a weekly basis either 40 mg/m^2 ^Cisplatin (group I; 16 patients) or 50 mg/m^2 ^paclitaxel (group II; 15 patients) concurrently with radiotherapy.

### Chemotherapy

Patients were randomized into two groups: group 1 treated with weekly cisplatin and group 2 treated with weekly paclitaxel. Group I patients received **w**eekly cisplatin 40 mg/m^2 ^given intravenously in 200 cc of D5-NSS over one hour. Premedication consisted of dexamethasone 8 mg IV, and a 5HT3-receptor antagonist as antiemetic with hydration for two hours before and after chemotherapy with D5-NSS at 150 cc/hour. Group II patients were treated with weekly paclitaxel 50 mg/m^2 ^given intravenously in 500 cc of D5W in a glass container over three hours. Premedication consisted of dexamethasone 8 mg IV, Benadryl 25 mg IV, Ranitidine 50 mg IV, and a 5HT3-receptor antagonist as antiemetic. Planned treatment was for an average of 5-6 cycles to coincide with the duration of external beam radiation and continued during brachytherapy.

### Radiation therapy

Radiation treatment consisted of external beam radiation to 40 Gy in 20 fractions using 15 MV photons and an anteroposterior pelvic field arrangement. This was followed by low-dose, or high-dose rate uterovaginal brachytherapy (UVB). Patients treated before 2001 received low-dose Cesium brachytherapy, but after 2001, eligible patients were treated with high-dose rate Iridium brachytherapy. Patients who had parametrial involvement also received a parametrial boost to the affected side. Median total dose to point A was 74 Gy (range: 66-92 Gy) for group I and 66 Gy (range: 40-98 Gy) for group II. Patients who had poor vaginal anatomy, or had prior hysterectomy were treated with an external radiation boost instead of uterovaginal brachytherapy to a median total central pelvic dose of 60.4 Gy (range: 60-66 Gy). The first HDR brachytherapy application was inserted no later than 7 days after completion of pelvic radiation.

### Baseline evaluation and follow-up

Tumor size was assessed clinically by two different examiners prior to, and following treatment. Initial work-up included a complete blood and platelet counts (CBC), creatinine (Cr), liver function tests (SGPT, Alk. P., Gamma GT). Computerized tomography (CT) scanning of the abdomen and pelvis, and chest x-ray (CXR). During treatment, patients had weekly CBC, Cr., and liver function tests. Patients who developed any allergic reactions during cisplatin and paclitaxel, were subsequently pretreated with dexamethasone 8 mg orally every 8 hours for three doses prior to the admission for chemotherapy. After completing therapy, patients had a repeat baseline examination and CT scan of the abdomen and pelvis. Later follow-up evaluation consisted of repeat pelvic examinations every 3 months for the first two years and every six months thereafter until progression or deaths. Pap smears, CXR, and CT scans of the abdomen and pelvis were obtained every 6 months.

### Endpoints ans statistical anlysis

The primary endpoints were treatment response, overall survival, and time to relapse. *Time to relapse *was defined from the date of entry into the study to the date of recurrence. Patients with *progressive disease *who never achieved complete response, were censored for relapse at the end of radiation therapy, which is approximately 2 months and half from the date of entry. *Disease free survival *was defined as alive with no evidence of disease at the time of last follow-up or dead without evidence of disease. *Survival *was calculated from the date of entry to the date of death or last follow-up. *Cumulative survival rates *was estimated by the Kaplan-Meier method and compared using the Logrank tests. Chi-square and the student' *t-*test were used for comparative analyses. Statistical significance was defined as p < 0.05. Data were analyzed using a SPSS program version 16.0. Patient lost to follow up were censored from the analysis at the time of their last follow up date. Treatment related toxicity was assessed as a secondary endpoint. Adverse events were graded according to the National Cancer Institute Common Toxicity Criteria grading system [36]. The incidence, severity, and causal relation to treatment of these events were compared between treatment groups. The study was conducted according to globally accepted standards of Good Clinical Practice and in agreement with the declaration of Helsinki and was approved by the Institutional Review Board (IRB). All patients have signed an informed consent form before their enrollement into the study.

## Results

Thirty one consecutive patients met the eligibility criteria and were enrolled; 16 patients were randomized to the cisplatin arm (group I) and 15 to the paclitaxel arm (group II). The patients' demographic characteristics are listed in **table **1. Age, parity, histology, presence of hydronephrosis, and lymph node involvement were not different in both groups. Only median tumor size was slightly larger for group II patients (6 cm) compared to that of group I patients (4.75 cm), but this was not statistically significant (p = 0.16).

**Table 1:** Patient characteristics. Group I received concurrent cisplatin and group II received concurrent paclitaxel.

	Group I	Group II
Number of patients	16 *	15 *
Median age (years)	56 (37-71) *	48 (38-80) *
Parity	4 (0-11) *	5 (2-9) *
Squamous cell pathology	13 (81%) *	14 (93%) *
Stage III-IVA	7 (44%) *	8(53%) *
Median Tumor size (cm)	4.75 (2.5-8) *	6 (3-11) *
Hydronephrosis	3 (19%) *	5 (33%) *
Positive pelvic lymph nodes	3 (19%) *	5 (33%) *
Enrolled as pelvic recurrence	2 (13%) *	5 (33%) *

* Shown in parentheses, are range or percentage values as applicable.

Treatment details are listed in **table **2. The mean number of chemotherapy cycles was comparable [4.6 ± 0.9 vs. 4.3 ± 1.3], with 87% and 80% of patients receiving ≥4 doses in groups I and II, respectively. The median dose to point A was slightly higher for group I patients (74 vs 66 Gy, p = 0.27). This is due to the fact that more patients in group II were enrolled as pelvic recurrences after surgery and received an external beam radiation boost instead of uterovaginal brachytherapy. In group I, only 3 patients (19%) did not receive UVB compared to 6 patients (40%) in group II (p = 0.84). The mean duration of radiation therapy was similar in both groups (53.1 ± 9.9 vs. 55.8 ± 9.0 days; p = 0.56). Treatment related acute toxicity is listed in **table **3. Both groups had comparable hematological toxicity, but more patients in group II had severe diarrhea (53% vs. 37%), and severe allergic reactions (40% vs. 6%). In two group II patients, chemotherapy had to be discontinued because of drug-related severe allergic reactions. Also, delay in chemotherapy was more common with group II than with group I patients (47% vs. 25%), but this difference was not statistically significant (p = 0.22).

**Table 2:** Chemotherapy and radiation therapy treatment parameters.

	Group I	Group II
Chemotherapy cycles	5 (3-6) *	5 (1-6) *
>4 cycles	14 (87%) *	12 (80%) *
EBRT dose	40 (40-66) *	40 (40-66) *
Dose to point A	33 (5-52) *	34 (0-58) *
No UV brachytherapy	3 (19%) *	6 (40%) *
Total dose to point A	75 (60-93) *	66 (40-98) *

Group I received concurrent cisplatin and group II received concurrent paclitaxel.

* Numbers represent number of patients with percentages when indicated, or median values with ranges (shown in parentheses).

EBRT = external beam radiotherapy; UV=uterovaginal

**Table 3:** Incidence and types of acute toxicity.

Toxicity endpoint	Group I	Group II
Leucopenia (grade 1-4)	3 (19%)	4 (27%)
All hematologic (grade 3-4)	2 (12%)	1 (7%)
Neurologic (grade I)	2 (12%)	0
Diarrhea (grade 3-4)	6 (37%)	8 (53%)
Allergic reactions	1 (6%)	6 (40%)
Delay in CT	4 (25%)	7 (47%)

Group I received concurrent cisplatin and group II received concurrent paclitaxel. Two patients in group II had to discontinue treatment in cycles 1 & 2 because of severe allergic reactions.

*One patient in group I developed an allergic reaction to metochlorpropramide given as antiemetic*.

There was a non-significant trend for more local and distant failure in group II. Seven patients of group I (44%) suffered tumor relapse [three locally and four outside the pelvis], while 8 group II patients (53%) developed tumor recurrence [three locally, two with distant metastasis, and three locally and distantly]. At 2 years, local control rates were 93% for group I and 70% for group II (p = ns).

**Figure 1:**
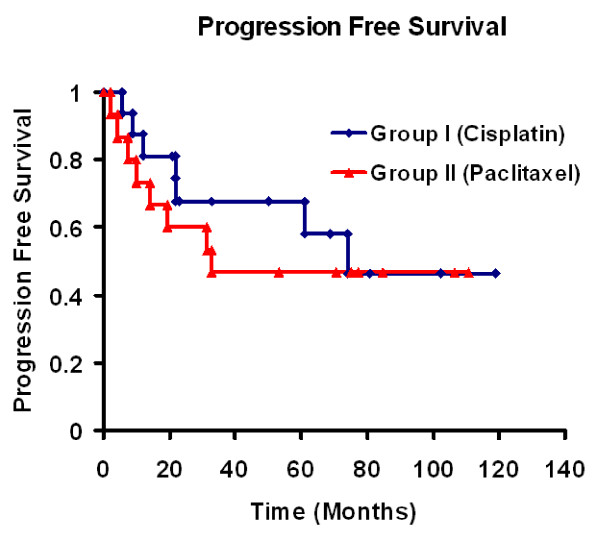
**Kaplan-Meier analysis of progression free survival per treatment group**.

**Figure 2:**
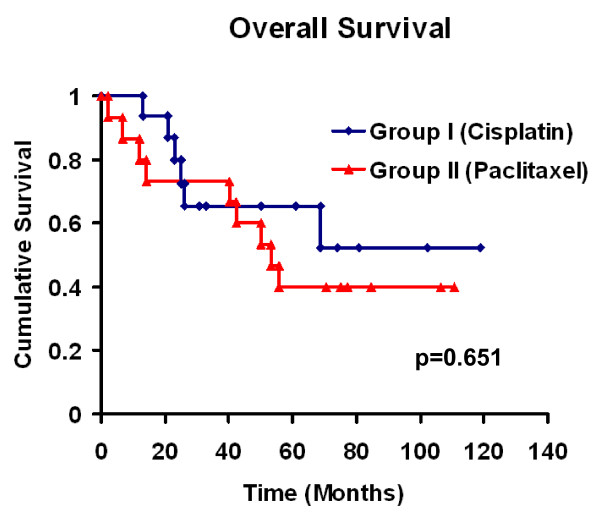
**Kaplan-Meier analysis of Overall survival per treatment group**.

Figure 1 shows progression free survival with no difference for both groups. The Median survival time was not reached for Group I and is 53 months for group II (Figure 2). At the end of the study 54% of group I *vs*. 42% of group II patients were still alive. The proportion of patients surviving at 2 and 5 years was 78% and 54% for group I *vs*. 73% and 42% for group II respectively (p = 0.651).

Several treatment and patient related factors were assessed for their prognostic significance. These included age, number of chemotherapy cycles, disease stage, presence of hydronephrosis, tumor size, delay in chemotherapy, radiation dose to point A, and the use of uterovaginal brachytherapy. None of these factors was found to have significant influence on disease free or overall survival (**table **4).

**Table 4:** Univariate analysis of several patient, tumor, and treatment parameters.

Factor	p-value
Age	0.29
Number of chemotherapy cycles	0.06
Stage	0.75
Hydronephrosis	0.93
Tumor size	0.16
Delay in Chemotherapy	0.22
Dose to Point A	0.27
HDR brachytherapy	0.84

*Only the number of chemotherapy cycles was borderline significant*.

## Discussion

This small phase II study provides a direct comparison between cisplatin and paclitaxel used as weekly concurrent chemotherapy with definitive radiation for advanced carcinoma of the cervix. Our data indicate that the overall response and progression free survival rates with the use of paclitaxel, which is the experimental arm, are not superior to those with cisplatin. In fact, there were non-significant trends for a higher relapse rate, higher gastrointestinal toxicity, and more allergic reactions in the concurrent paclitaxel group. Taken together, these results indicate that paclitaxel does not provide any clinical advantage over the current standard of concurrent cisplatin in CTRT for patients with advanced cervical carcinoma.

Although many prospective studies had shown that CTRT with cisplatin-based chemotherapy clearly improve the outcome of patients with carcinoma of the cervix, many patients treated on these protocols continue to fail in the pelvis and at distant sites [6,16,22,23]. In addition, one intergroup study using weekly concurrent cisplatin with radiotherapy for patients with carcinoma of the cervix could not demonstrate a beneficial effect of CTRT over standard RT alone [24]. This non-superiority finding was attributed to many factors like possible enrollment of patients with paraaortic lymph nodes, and an imbalance among randomization groups for known prognostic factors such as anemia [22,37]. These facts have lead many groups to investigate other drugs for CTRT like paclitaxel in an attempt to improve on what can be achieved by concurrent cisplatin [31,38-44]. In all these studies paclitaxel was used in conjunction with either cisplatin (4/7 studies) or carboplatin (3/7 studies) but was never used alone for CTRT. The majority of these studies was phase I (4/7 studies), with one study being a combined phase I/II study conducted by the GOG [42]. The number of patients enrolled in these studies varied between 8 and 35 patients and the rates of progression free survival ranged between 39 and 88%. The dose limiting toxicity was primarily neutropenia in 4 studies [38,41,42,44] or diarrhea [31,39,40]. In our phase II study reported here, we enrolled 31 patients and progression free survival was 61% for the cisplatin arm and 63% for the paclitaxel arm with severe grade III diarrhea being the most common toxicity (37% and 54% for the cisplatin and paclitaxel groups, respectively). These data are in agreement with what other groups have reported and do not suggest that paclitaxel provides any advantage in outcome or toxicity over the current standard using cisplatin. This finding is in line with what was found in a larger study by the GOG which also compared concurrent single agent CTRT consisting of either weekly cisplatin or protracted 5-fluorouracil (5-Fu) infusion. The results of that study showed no superiority of the experimental 5-Fu arm and the study was prematurely closed [45].

There are many studies that investigated other experimental protocols for concurrent CTRT in advanced carcinoma of the cervix using various chemotherapy regimens such as 5-Fu, epirubicin, 5-Fu with mitomycin C, hydroxyurea, gemcitabine, carboplatin, tirapazamine, topotecan, or vinorelbine [46-64]. Few of these drugs were tested in randomized trials like 5-Fu, epirubicin, hydroxyurea, mitomycin and gemcitabine, as single agents or in combination [46-48,53]. Others have only been tested in phase I/II studies and some of them have shown promising results. Perhaps the most promising and most studied drugs of this group are gemcitabine, tirapazamine, and topotecan. In a phase II randomized study by Dueñas-Gonzalez et al, patients with stage IB2-IIB disease were randomized to cisplatin or cisplatin plus gemcitabine and concurrent radiation therapy, followed by radical hysterectomy 4 weeks later. The complete pathologic response rate was higher in the cisplatin plus gemcitabine arm compared to the cisplatin alone arm (75% vs. 55%, respectively; p = 0.02), but gastrointestinal and hematologic toxicities were significantly lower in the cisplatin-alone arm [53]. A phase III randomized trial testing this combination for definitive CTRT in stages IIB to IVA disease, has completed accrual but results are not yet available. Similarly, encouraging results have been obtained in phase I studies using cisplatin-combination CTRT with either topotecan or tirapazamine [60-62]. Several phase I/II studies are currently investigating the combination cisplatin-topotecan and GOG trial 0219 is testing the added value of tirapazamine to cisplatin for CTRT in carcinoma of the cervix.

The rate of gastrointestinal (GI) toxicity in our study manifesting as severe diarrhea, was high in both arms although slightly higher in the paclitaxel arm. In addition there were more severe allergic reactions in the paclitaxel arm and in 2 patients, chemotherapy had to be discontinued due to the severity of these allergic reactions, and in general, more chemotherapy delays were encountered in this group. It is difficult to compare this toxicity pattern with other studies from the literature, because none of these studies used either paclitaxel or cisplatin alone for CTRT, instead they used both drugs in combination with various dose-administration schedules. However, one could note that in at least 3 of the phase I studies that included paclitaxel, severe diarrhea was the limiting toxicity which agrees with our findings [31,39,40]. It is of concern that this difference in toxicity between our treatment groups with the disruption and delays in chemotherapy delivery in group II, could have negatively affected this group's outcome. This is particularly important because this group was also at a relative disadvantage regarding tumor bulk (larger median tumor size) and a smaller number of those patients could benefit from UV brachytherapy (9/15 vs. 13/16 for group I) due to anatomical constraints. However, because of the small size of the study it was not possible to fully evaluate the influence of these factors either separately or all combined.

A total of 10 patients (32%) from both treatment groups developed systemic metastases, a rate consistent with what is reported in the literature, and which emphasizes that the risk of distant recurrence remains a major concern for these high risk patients. Taken as individual studies, data from the various CTRT trials have not consistently shown a reduction in distant metastases (DM) in patients receiving systemic chemotherapy when it was primarily given as a radiosensitizer [6-16,23,24]. However, when these data were analyzed together, two metaanalyses found a positive effect of concurrent CTRT on distant recurrence [65,66]. Among the studies that used platinum-based chemotherapy, only the radiation therapy and oncology group (RTOG) 90-01 study showed a significant effect of CT on the reduction of DM at both 5 and 8 years of follow-up [7,23]. It is of interest to note, that in that study, chemotherapy was given as full cycles of cisplatin and 5-Fu during the course of RT, and the dose of cisplatin was highest compared to what was used in the other studies (75 mg/m^2 ^vs. 60, 50, or 40 mg/m^2^). Among the studies with noncisplatin-based chemotherapy, only the study reported by Wong et al. showed a significant impact on DM [14]. In that trial, CT consisted of epirubicin as a single agent for concurrent CTRT followed by adjuvant therapy with the same drug for 5 cycles. It remains unclear what are the key factors that made the experimental arm in these two particular studies effective against DM. One could speculate that the delivery of full cycle and higher doses of chemotherapy, and/or the use of planned adjuvant chemotherapy could, in theory, better address the risk of systemic recurrence. However, this remains speculative until it is demonstrated in controlled randomized studies, and unfortunately, to our knowledge, there are no such studies currently in progress. The only available information comes from retrospective studies and there are some which critically examined this question and reported similar findings. In a single institution study, Kim et al. reported a comparison between 2 well balanced groups of patients with stage IB-II carcinoma of the cervix who were treated by concurrent CTRT with or without 3 additional cycles of adjuvant platinum (cisplatin or carboplatin)-5Fu chemotherapy [67]. The authors found no effect of adjuvant chemotherapy on the incidence of distant metastases or distant nodal relapses. They also found that adjuvant chemotherapy was relatively difficult to complete, with only 63% of the patients receiving all 3 cycles, and those in the adjuvant group experienced a higher rate of late grade III-IV rectal complications. Similar results were published by Lee et al. on patients receiving adjuvant CTRT after radical hysterectomy and treated either by 3 additional cycles of cisplatin-5Fu or no additional therapy [68]. Although these two studies are not prospective or randomized trials, they still indicate that the routine use of adjuvant cisplatin-based chemotherapy may not be the best approach to address the risk of distant relapse in this patient population.

In summary, these data show that concurrent chemoradiation for advanced cervical cancer using weekly paclitaxel was not superior to concurrent cisplatin and was possibly associated with more severe gastrointestinal toxicity and more allergic reactions. Progression free survival was equivalent with both drugs and failure at distant sites remains high in both groups, which may indicate the need for additional effective therapy.

## Competing interests

The study was partially supported by Bristol Myers Squibb. FG, AS, AK, and MS have received travel funds from Bristol Myers Squibb

## Authors' contributions

FG contributed to the study design, patient treatment, evaluation, and was the leading writer of the manuscript. AS, AK, and MA contributed to the study design and manuscript writing and review. MS performed the statistical analysis and contributed to the review. MS is the leading investigator and contributed to patient enrollment, follow-up and to the manuscript writing and review. All authors read and approved the final manuscript.
